# Intramedullary Nail versus Dynamic Compression Plate Fixation in Treating Humeral Shaft Fractures: Grading the Evidence through a Meta-Analysis

**DOI:** 10.1371/journal.pone.0082075

**Published:** 2013-12-16

**Authors:** JianXiong Ma, Dan Xing, XinLong Ma, Feng Gao, Qiang Wei, HaoBo Jia, Rui Feng, JingTao Yu, Jie Wang

**Affiliations:** 1 College of Precision Instrument and Opto-Electronics Engineering, Tianjin University, Tianjin, China; 2 Department of Orthopaedics, Tianjin Medical University General Hospital, Tianjin, China; 3 Department of Orthopaedics Institute, Tianjin Hospital, Tianjin, China; University Hospital Heidelberg, Germany

## Abstract

There is a debate regarding the choice of operative intervention in humeral shaft fractures that require surgical intervention. The choices for operative interventions include intramedullary nailing (IMN) and dynamic compression plate (DCP). This meta-analysis was performed to compare fracture union, functional outcomes, and complication rates in patients treated with IMN or DCP for humeral shaft fractures and to develop GRADE (Grading of Recommendations, Assessment, Development, and Evaluation)-based recommendations for using the procedures to treat humeral shaft fractures. A systematic search of all the studies published through December 2012 was conducted using the Medline, Embase, Sciencedirect, OVID and Cochrane Central databases. The randomized controlled trials (RCTs) and quasi-RCTs that compared IMN with DCP in treating adult patients with humeral shaft fractures and provided data regarding the safety and clinical effects were identified. The demographic characteristics, adverse events and clinical outcomes were manually extracted from all of the selected studies. Ten studies that included a total of 448 patients met the inclusion criteria. The results of a meta-analysis indicated that both IMN and DCP can achieve similar fracture union with a similar incidence of radial nerve injury and infection. IMN was associated with an increased risk of shoulder impingement, more restriction of shoulder movement, an increased risk of intraoperative fracture comminution, a higher incidence of implant failure, and an increased risk of re-operation. The overall GRADE system evidence quality was very low, which reduces our confidence in the recommendations of this system. DCP may be superior to IMN in the treatment of humeral shaft fractures. Because of the low quality evidence currently available, high-quality RCTs are required.

## Introduction

Fractures of the humeral shaft are commonly encountered in orthopedic clinics, and these fractures make up 1.31 to 3% of all fractures [1]. The treatment approaches for these injuries continue to evolve as advances are made in both non-operative and operative management[2]. The humeral shaft is covered with muscles and is well-vascularized. A slight malunion is functionally tolerated. It is generally agreed that the majority of humeral shaft fractures are best treated non-operatively, but there are indications for primary or secondary operative treatment in some situations[3]–[5]. Non-operative or conservative treatment may involve the use of casts or functional braces. In cases associated with severe complications, an operative intervention is preferred. The encouraging outcomes that have been demonstrated with recent advances in internal fixation techniques and instrumentation have led to an expansion of surgical indications for humeral shaft fractures and new debates regarding the procedure of choice.

Surgical treatment is generally indicated in patients in whom there is a failure to maintain stable alignment and reduction at the fracture site and in the patients with severe segmental fractures, open fractures, or fractures associated with bilateral fractures, forearm fractures on the same side, poly-trauma, progressive neurological deficits, vascular injury or floating shoulder or elbow [6], [7]. The options for the commonly used surgical treatment of humeral shaft fractures include intramedullary nailing (IMN) and dynamic compression plate (DCP), which offer good clinical outcomes. At present, both of these surgical approaches are used to treat humeral shaft fractures. Both techniques have certain mechanical and anatomical advantages and disadvantages. Plating with stable fixation and direct visualization, which is known to provide an accurate anatomic reduction and protection of the radial nerve, can reduce the risk of malunion but requires wide intraoperative exposure associated with soft-tissue stripping. Continuous innovation in the design of IMN has ensured the clinical application of intramedullary fixation in treating humeral shaft fractures. Several studies recommended IMN as a standard surgical method through antegrade or retrograde nailing [8]–[10]. IMN has the advantage of closed insertion techniques, intact periosteal blood supply, and load-sharing mechanical properties. The IMN can reduce the effects of stress shielding at the fracture site and lower the incidence of refracture after implant removal. One primary complication of antegrade IMN is rotator cuff impairment, which might lead to shoulder impingement and the restriction of shoulder motion. Iatrogenic comminution of the fracture site during retrograde reaming and iatrogenic damage of the radial nerve during ante grade nailing are common complications during the operation.

Several randomized controlled trials (RCTs) or quasi-RCTs have been conducted to compare IMN with DCP in treating humeral shaft fractures. There is controversy over which of the two procedures leads to superior results and better clinical outcomes. There is no consensus as to whether IMN or DCP is the optimal treatment. The purpose of this meta-analysis is to evaluate the evidence from the RCT and quasi-RCT studies that compared the safety and efficacy of IMN and DCP for treating patients with humeral shaft fractures and to develop GRADE (Grading of Recommendations, Assessment, Development, and Evaluation)-based recommendations for using the procedures to treat humeral shaft fractures [11], [12].

## Materials and Methods

### Search strategy and eligibility criteria

To assemble all of the relevant published studies, PRISMA compliant searches of MEDLINE, EMBASE, ScienceDirect, OVID, the Cochrane CENTRAL databases and Google scholar were performed for all the peer-reviewed studies published through February 2013 that compared KP to VP for treating OVCF. The following search terms were used to maximize the search specificity and sensitivity: humeral fractures, fracture fixation, intramedullary nails, and bone plates. Broad MeSH terms and Boolean operators were selected for each database search. Secondary investigations into the unpublished literature were performed by searching the WHO International Clinical Trials Registry database, Current Controlled Trials, European Federation of National Associations of Orthopaedics and Traumatology and British Orthopaedic Association Annual Congress, and the ISTP database.

The complete articles found by the above search methodology were retrieved and assessed for the inclusion/exclusion criteria outlined in Table 1. The references from relevant articles were also reviewed. We made no restrictions on the publication language.

**Table 1:** Inclusion/exclusion criteria.

Inclusion criteria	Exclusion criteria
Randomized control trials (RCTs)	Case reports
Quasi-randomized control trials (qRCTs)	Cadaver or model studies
Age 18 years or older	Biomechanical studies
Humeral shaft fractures	Fractures within the proximal and distal end of the humerus
Comparing DCP and IMN	Pathological fractures/metastatic malignant disease
	Shoulder degenerative disease or ipsilateral shoulder injury
	Refractures of the humerus

IMN: intramedullary nail; DCP: dynamic compression plate.

### Study selection

Two reviewers (MJX and XD) independently screened the titles and abstracts for the eligibility criteria. The full text of the studies that potentially met the inclusion criteria were read, and the literature was reviewed to determine the final inclusion. We resolved disagreements by reaching a consensus through discussion.

### Date extraction

Two of the authors (MJX and XD) independently extracted the data from each full-text report using a standard data extraction form. The data extracted from studies included the title, authors, year of publication, study design, sample size, population, age, gender, type of interventions, surgical procedures, duration of follow-up, and outcome parameters. The corresponding authors of the included studies were contacted to obtain any required information that was missing. The extracted data were verified by MXL.

### Outcomes

The clinical outcomes included: fracture union, iatrogenic radial nerve injury, intraoperative fracture comminution, infection, shoulder impingement, restriction of shoulder range of movement, implant failure, American Shoulder and Elbow Surgeons (ASES) score.

### Assessment of methodological quality

According to Cochrane Handbook for Systematic Reviews of Interventions 5.0, the risk of bias of the included studies was assessed by two authors (MJX and XD) independently. Disagreements were resolved by discussion. A third author (MXL) was the adjudicator when no consensus was achieved. We applied the “assessing the risk of bias” table, which include the following key domains: adequate sequence generation, allocation of concealment, blinding, incomplete outcome data, free of selective reporting and free of other bias.

### Data analysis

We performed all of the meta-analyses with the Review Manager software (RevMan Version 5.1; The Nordic Cochrane Center, The Cochrane Collaboration, Copenhagen, Denmark). For continuous outcomes, such as the ASES score, the means and standard deviations were pooled to a weighted mean difference (WMD) and a 95% confidence interval (CI). The risk ratios (RRs) and 95% confidence intervals (CIs) were used to evaluate the dichotomous outcomes, such as the incidence of bone cement leakage or adjacent fractures. The inverse variance and Mantel-Haenszel techniques were used to combine the separate statistics. A P-value <0.05 was considered statistically significant. The statistical heterogeneity was assessed using Q statistics. A fixed-effects (inverse variance) model was used when the effects were assumed to be homogenous (P>0.05). P<0.05 implied statistical heterogeneity, and a random effects model was used in those circumstances.

Publication bias was assessed visually using a funnel plot and statistically using Begg funnel plots and Egger's bias test using STATA 12.0 software (Statacorp, college station, Tex), which measures the degree of funnel plot asymmetry [13], [14]. The Begg adjusted rank correlation test was used to assess the correlation between the test accuracy estimates and their variances. The deviation of Spearman's rho values from zero provides an estimate of the funnel plot asymmetry. Positive values indicate a trend towards higher levels of test accuracy in studies with smaller sample sizes. Egger's bias test detects funnel plot asymmetry by determining whether the intercept deviates significantly from zero in a regression of the standardized effect estimates against their precision values.

### Evidence synthesis

The evidence grade was determined using the guidelines of the GRADE (Grading of Recommendations, Assessment, Development, and Evaluation) working group [12]. The GRADE system uses a sequential assessment of the evidence quality followed by an assessment of the risk-benefit balance and a subsequent judgment on the strength of the recommendations. The evidence grades are divided into the following categories: (1) high, which indicates that further research is unlikely to alter confidence in the effect estimate; (2) moderate, which indicates that further research is likely to significantly alter confidence in the effect estimate and may change the estimate; (3) low, which indicates that further research is likely to significantly alter confidence in the effect estimate and to change the estimate; and (4) very low, which indicates that any effect estimate is uncertain. Study limitations[15], results inconsistency[16], indirectness[17], imprecision[18] and publication bias[19] may lower the grade of the quality of evidence. The reasons for increasing the quality of evidence include a large effect, presentation of a dose-response gradient and plausible confounders that would decrease an apparent treatment effect[20]. As recommended by the GRADE working group, the lowest evidence quality for any of the outcomes was used to rate the overall evidence quality. The evidence quality was graded using the GRADEpro Version 3.6 software. The strengths of the recommendations were based on the quality of the evidence.

## Results

### Search results

**Figure 1:**
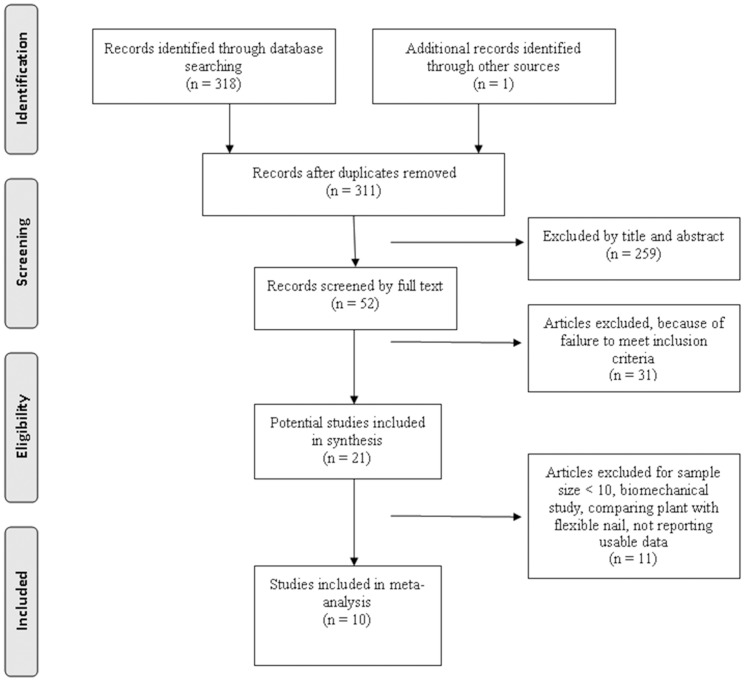
The study selection and inclusion process.

A total of 319 titles and abstracts were preliminarily reviewed, and 10 studies eventually satisfied the eligibility criteria (Fig. 1). These studies included 8 RCTs[8], [21]–[27] and 2 quasi-RCTs [28], [29]. One of the 10 studies was published only in abstract form [27]. All of the included studies compared IMN and DCP in the treatment of humeral shaft fractures.

### Quality assessment

**Figure 2:**
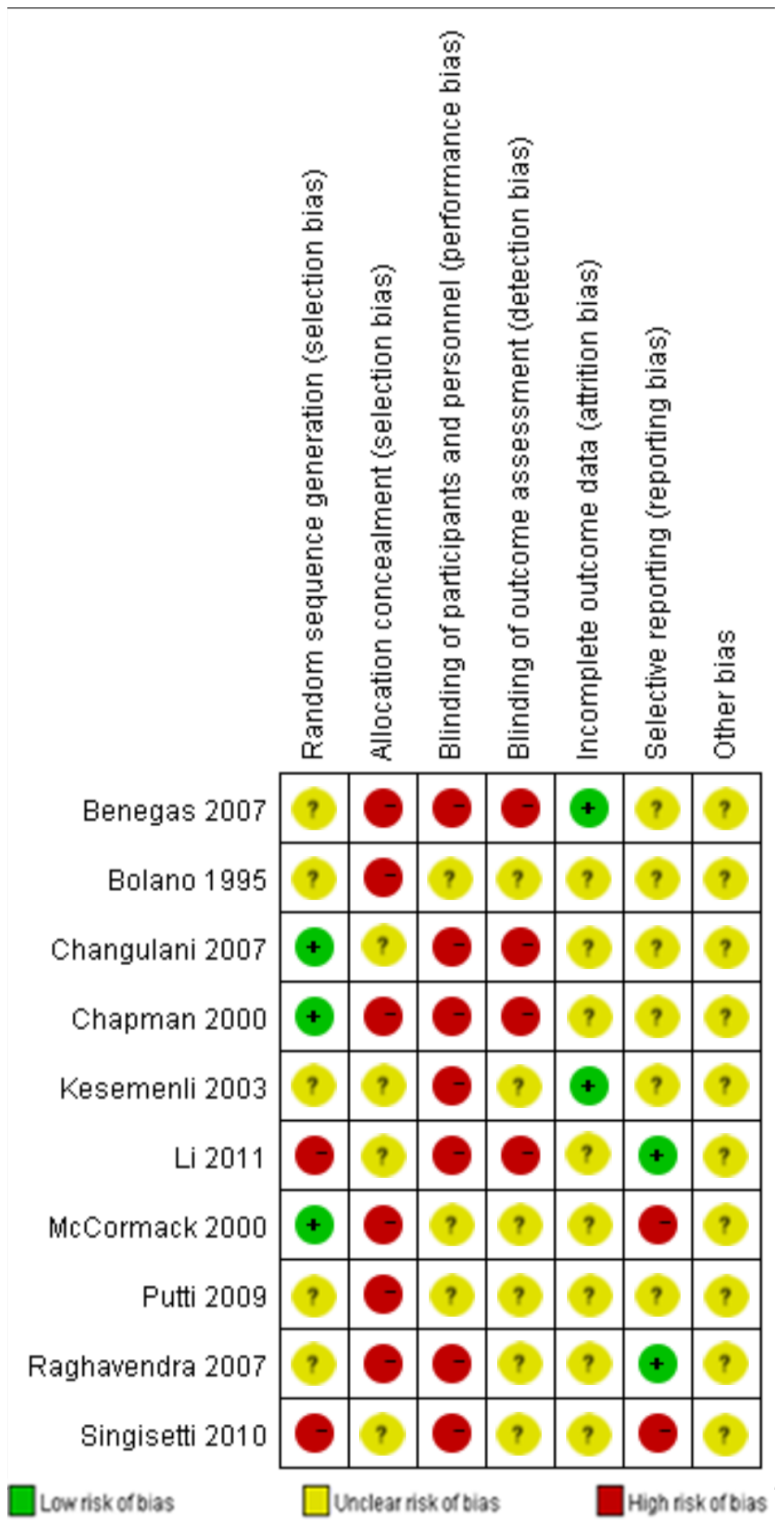
Methodological quality of the included studies. This risk of bias tool incorporates the assessment of randomization (sequence generation and allocation concealment), blinding (participants, personnel and outcome assessors), completeness of the outcome data, selection of the outcomes reported and other sources of bias. The items were scored with ‘yes’, ‘no’, ‘unsure’.

**Figure 3:**
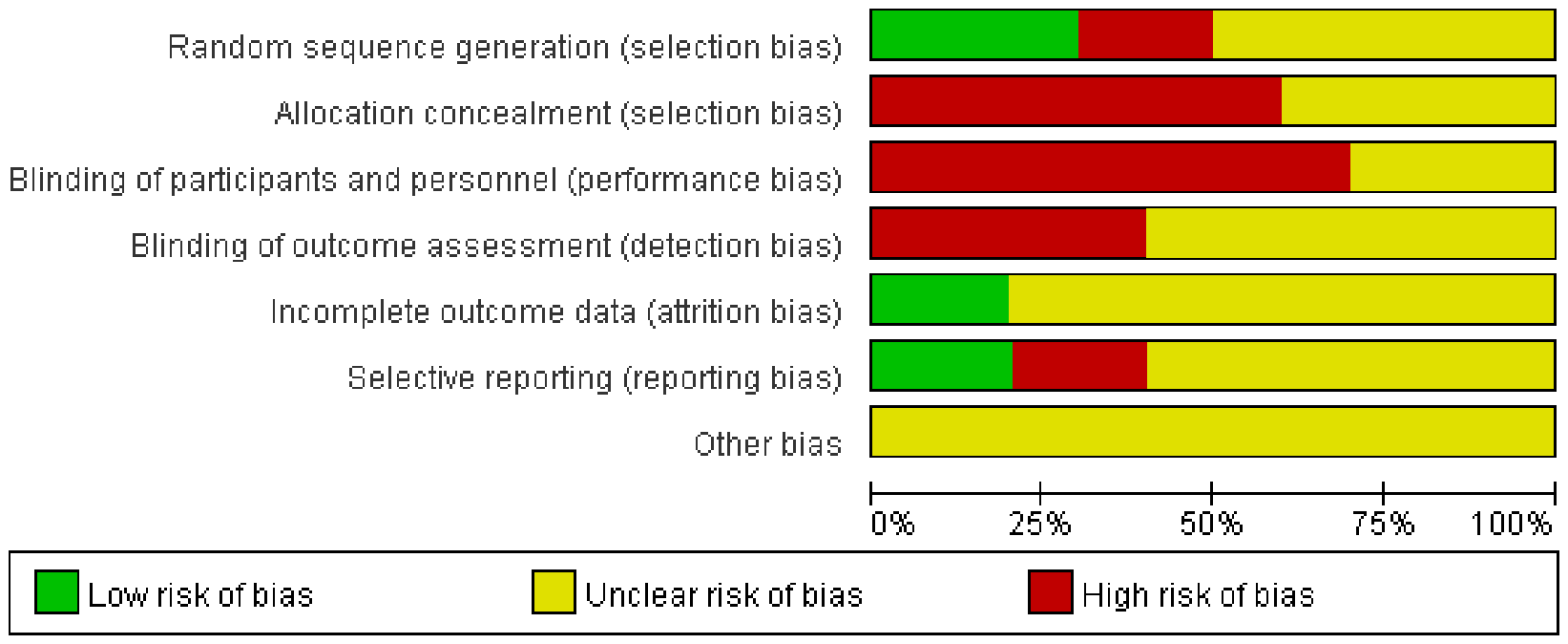
Risk of bias. Each risk of bias item is presented as the percentage across all the included studies, which indicates the proportion of different levels of risk of bias for each item.

Among the 10 included studies, the methods of randomization of 8 RCTs[8], [21]–[27] were not clear. The patients of the 2 quasi-RCTs[28], [29] were randomly allocated by odd and even hospital numbers. Three studies used sealed envelopes for allocation concealment. None of the included studies reported blinding to the surgeons, participants or assessors. None of the included studies reported whether or not they received grants in support of their research. None of the studies mentioned whether an “intention-to-treat” analysis was performed. All the included studies had a high risk of bias. The methodological quality of the included studies is presented in Fig. 2. Judgments regarding each risk of bias item are presented as percentages across all the included studies in Fig. 3.

### Demographic characteristics

The demographic characteristics of the studies included are summarized in Table 2. In total, 8 RCTs and 2 quasi-RCTs with 448 total patients were eligible for inclusion. The individual sample sizes ranged from 25 to 84 patients. A total of 224 patients underwent IMN, and the other 224 patients received a DCP. The one study[27] published in only abstract form did not state the genders and ages of the patients. All the patients were skeletally mature in the other nine studies. The percentage of males ranged from 48.8%[23] to 94.1%[21] in the nine studies. There were 2 studies performed in the USA, 2 in India, 2 in the UK, and 1 each in China, Turkish, Canada, and Brazil. All of the included studies had defined eligibility criteria. The indications for surgery was different among the studies, including both open and closed fractures and primary surgical treatment as well as the early failure of initial conservative treatment. Three studies[21], [28], [29] recruited patients with only closed fractures, and 5 studies[8], [22], [23], [25], [26] included patients with open and closed fractures. Whether the fractures were open or closed was not mentioned in two studies [24], [27]. Antegarde nailing was performed in 8 studies [8], [21]–[24], [26], [28], [29]. Only one study[25] conducted a trial with a locked nail that was later changed to a retrograde nail.

**Table 2:** Characteristics of the included studies.

Study	Year	Country	Study design	Sample size (IMN)	Sample size (DCP)	Mean age (IMN)	Mean age (DCP)	Gender (IMN)	Gender (DCP)	Follow-up (mo)	Conflicts of interest
Changulani et al.[8]	2007	India	RCT	23	24	39±12	35±11.5	20/3	19/5	14.3	N/S
Chapman et al.[23]	2000	USA	RCT	38	46	33 (18–70)	34 (18–83)	26/12	25/21	13	N/S
Kesemenli et al.[24]	2003	Turkish	RCT	33	27	42 (21–61)	33 (19–47)	43/17		42	N/S
McCormack et al.[25]	2000	Canada	RCT	21	23	40 (19–82)	49 (20–81)	13/8	15/8	14.3	N/S
Bolano et al.[27] (abstract)	1995	USA	RCT	14	14	NA	NA	NA	NA	NA	N/S
Putti et al.[21]	2009	UK	RCT	16	18	36 (23–84)	39 (22–65)	32/2		>24	N/S
Benegas et al.[22]	2007	Brazil	RCT	14	11	36.4 (19–75)	42.2 (19–71)	13/1	7/4	21	N/S
Singisetti et al.[29]	2010	UK	qRCT	25	20	18–63		35/10		12	N/S
Li et al.[28]	2011	China	qRCT	22	23	39.9±11.3	35.7±10.9	16/6	16/7	>12	N/S
Raghavendra et al.[26]	2007	India	RCT	18	18	40.2 (18–70)	40.8 (22–70)	15/3	17/1	>12	N/S

IMN: intramedullary nailing; DCP: dynamic compression plate; RCT: randomized control trial; NA: not available; mo: months.

### Outcomes analysis

**Figure 4:**
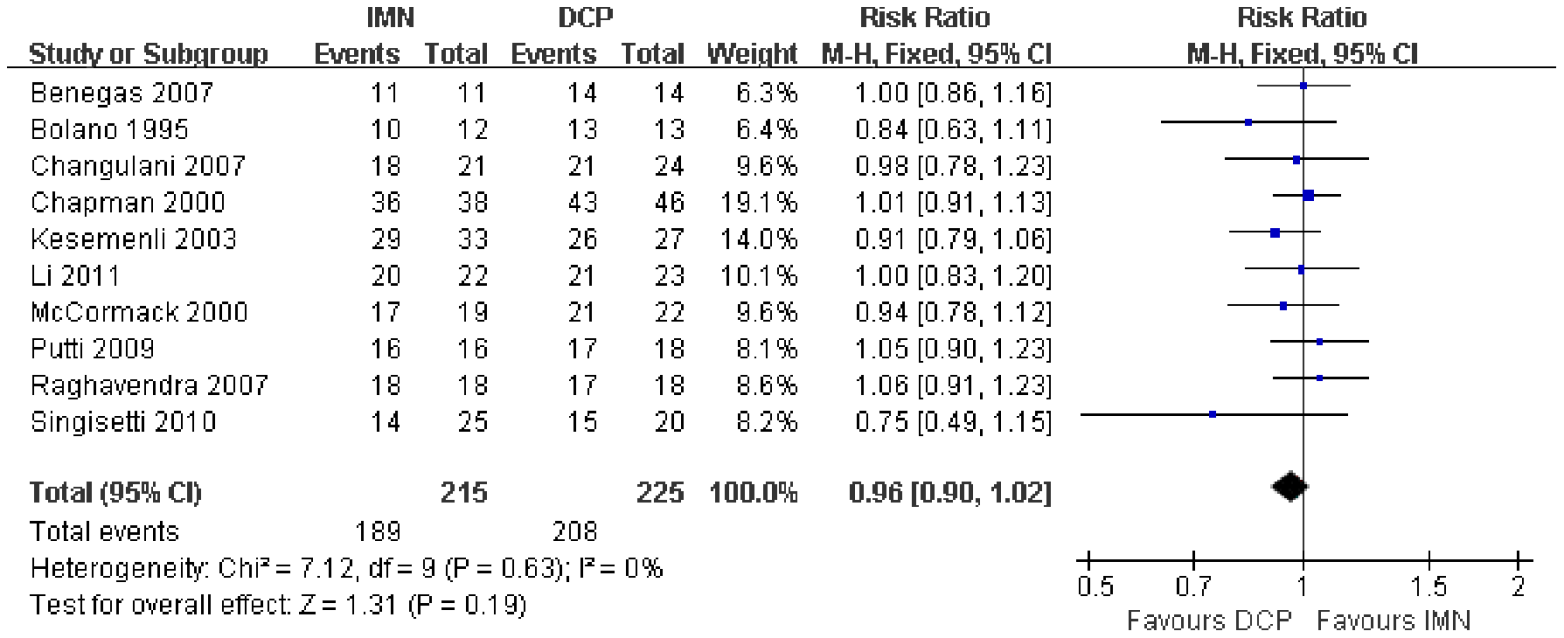
Forest plot for the risk ratio (RR) estimate for fracture union. RR = −0.96 (95% CI, 0.90–1.02).

**Figure 5:**
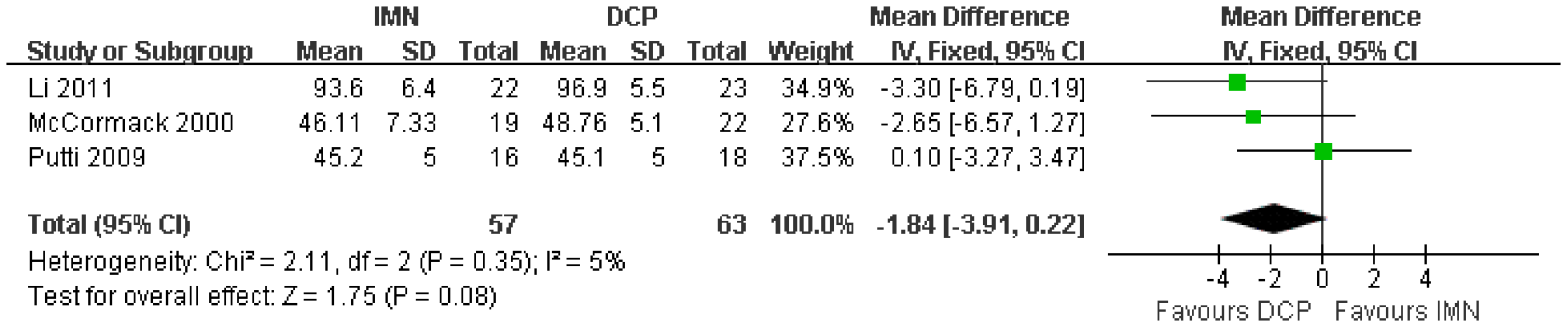
Forest plot for the weighted mean difference (WMD) estimate for ASES score. WMD = −1.84 (95% CI, −3.91–0.22).

**Figure 6:**
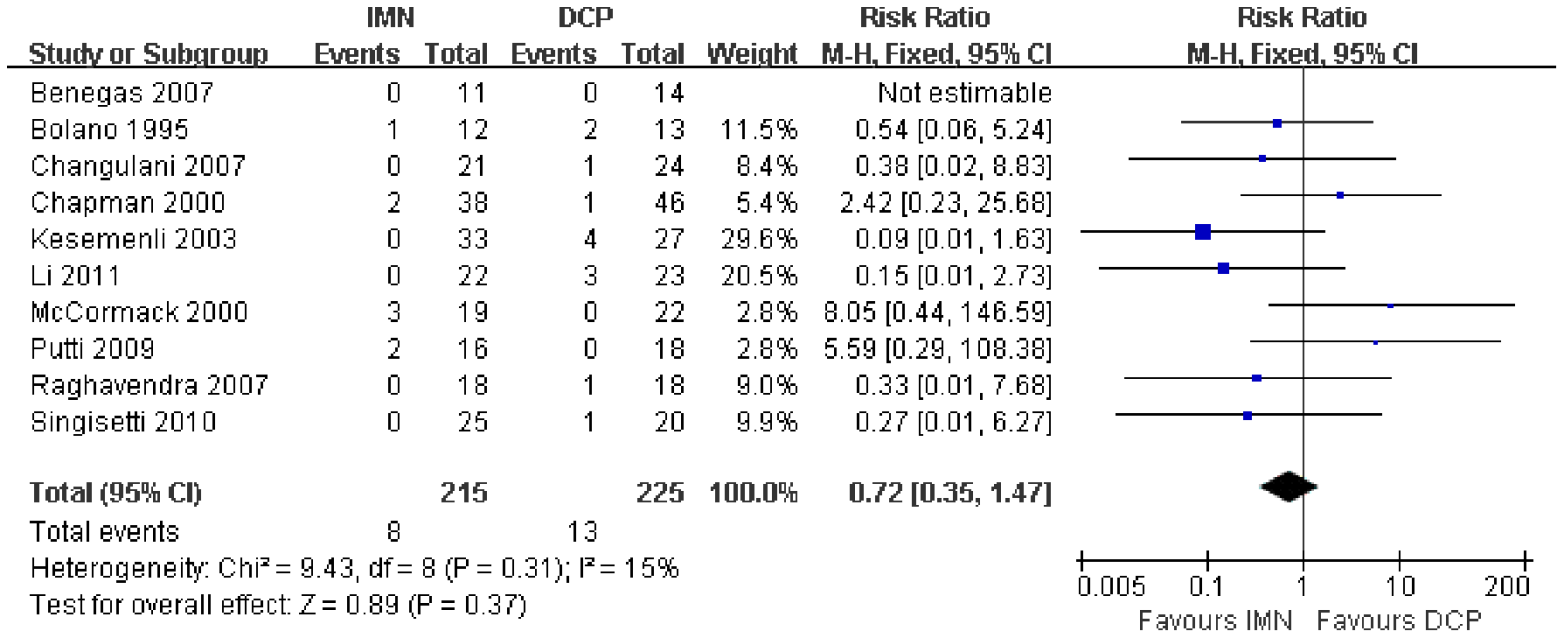
Forest plot for the risk ratio (RR) estimate for iatrogenic redial nerve injury. RR = 0.72 (95% CI, 0.35–1.47).

**Figure 7:**
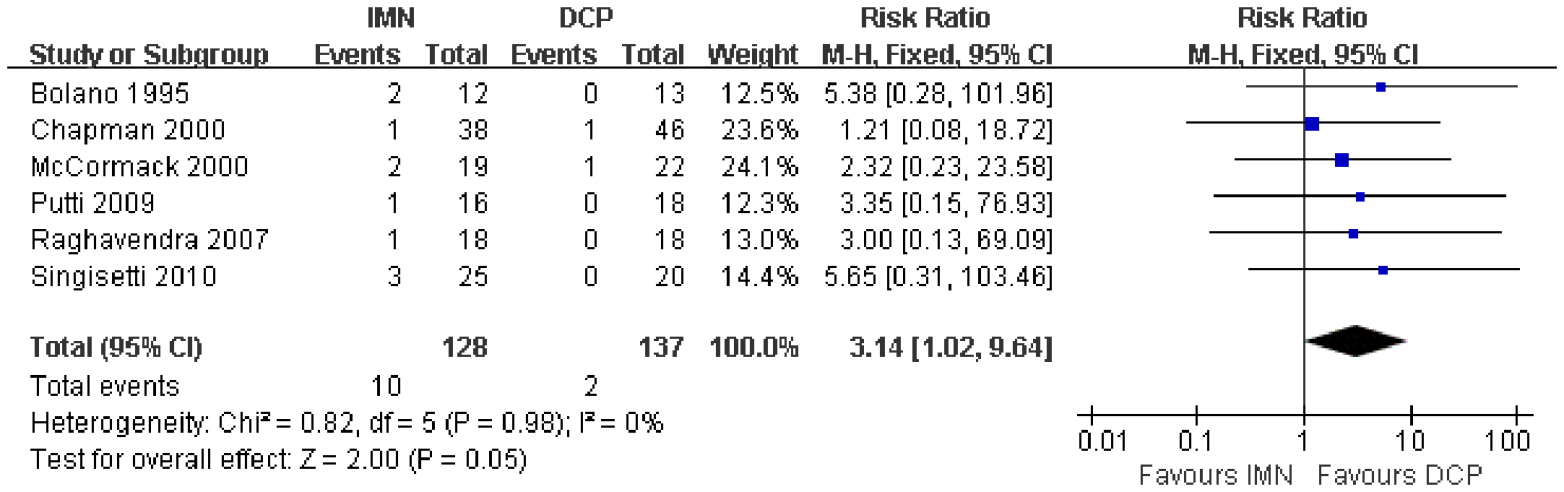
Forest plot for the risk ratio (RR) estimate for the rate of intraoperative fracture comminution. RR = 3.14 (95% CI, 1.02–9.64).

**Figure 8:**
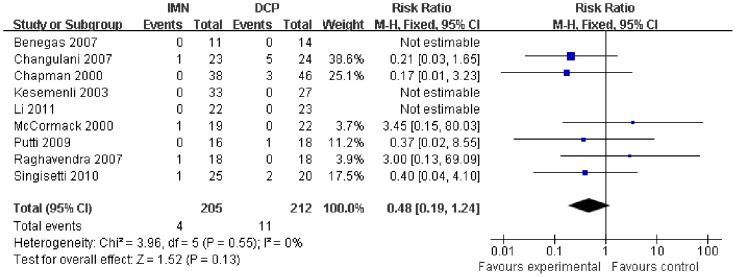
Forest plot for the risk ratio (RR) estimate for infection. RR = 0.48 (95% CI, 0.19–1.24).

**Figure 9:**
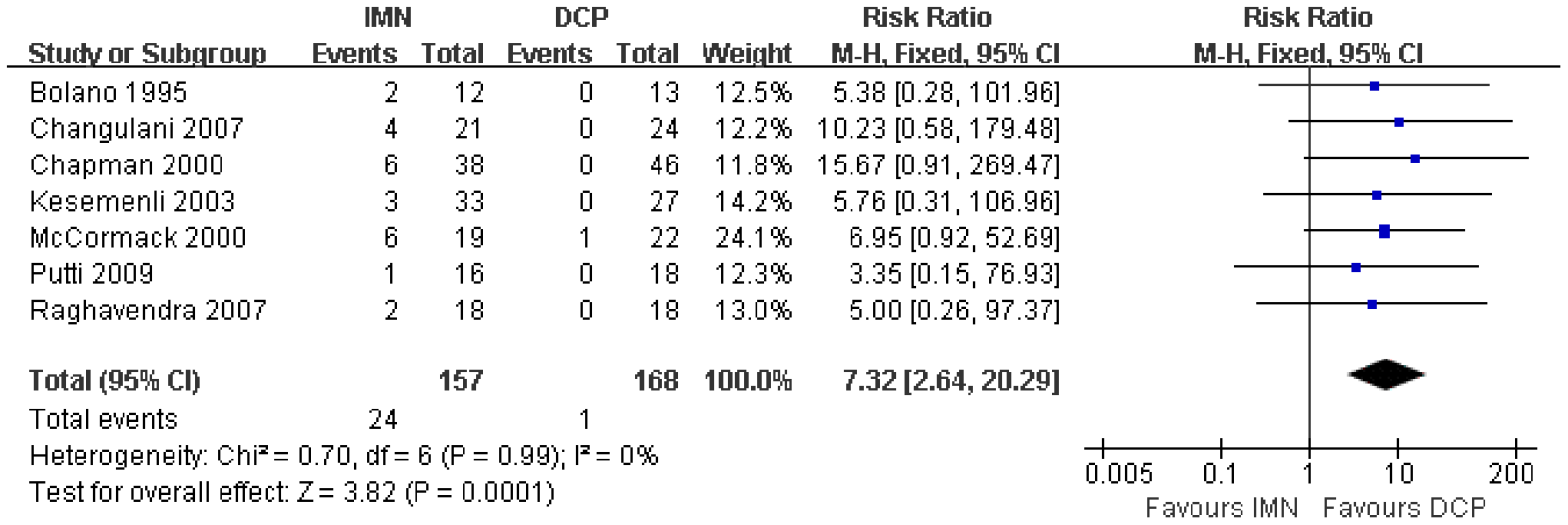
Forest plot for the risk ratio (RR) estimate for shoulder impingement. RR = 7.32 (95% CI, 2.64–20.29).

**Figure 10:**
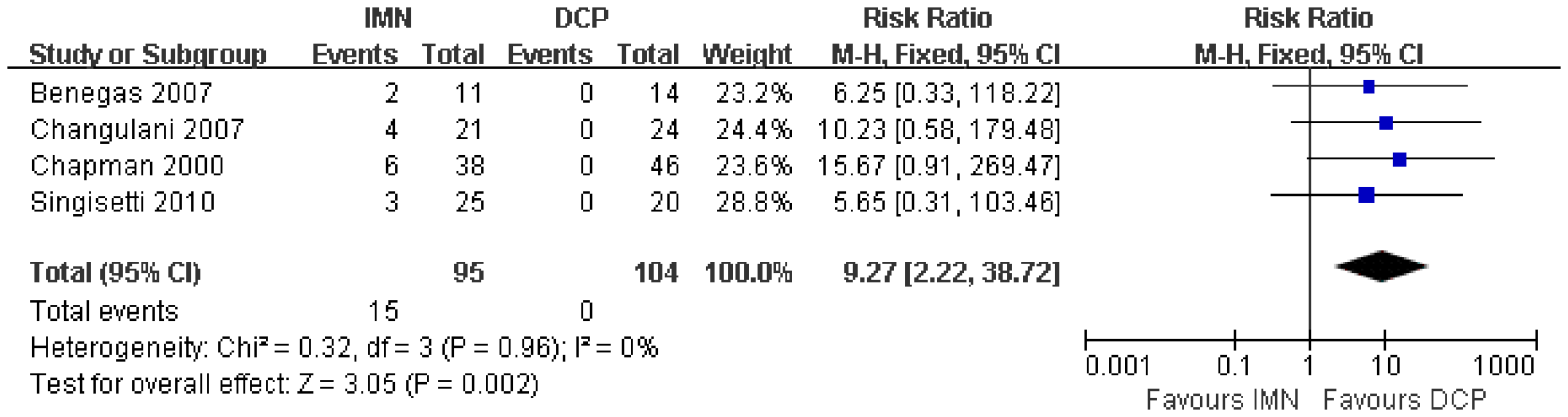
Forest plot for the risk ratio (RR) estimate for the incidence of restriction of shoulder range of movement. RR = 9.27 (95% CI, 2.22–38.72).

**Figure 11:**
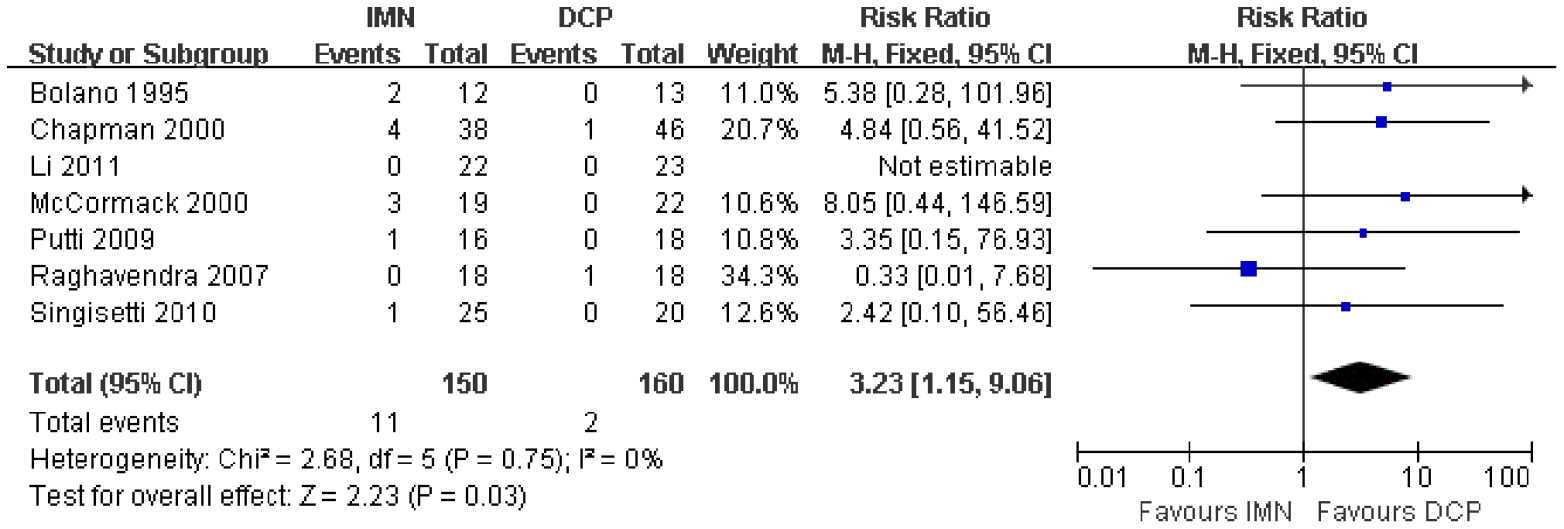
Forest plot for the risk ratio (RR) estimate for the incidence of implant failure. RR = 3.23 (95% CI, 1.15–9.06).

**Figure 12:**
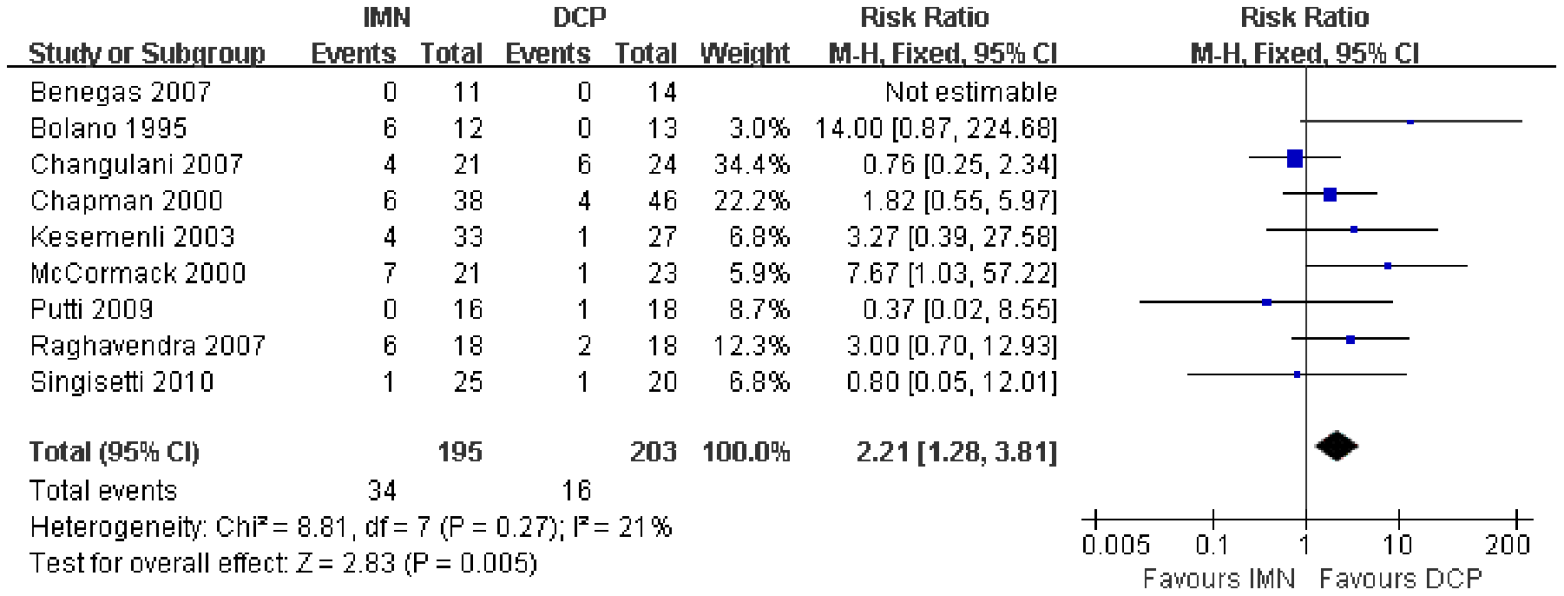
Forest plot for the risk ratio (RR) estimate for the incidence of re-operation. RR = 2.21 (95% CI, 1.28–3.81).

In all the studies providing comparisons of fracture union between IMN and DCP, the pooled RR was −0.96 (95% CI: 0.90–1.02). There was no significant difference between the groups (Fig. 4). Only 3 trials[21], [25], [28] reported the ASES score after the operation. There was no statistically significant difference between the two groups (WMD = −1.84 95% CI: −3.91–0.22) (Fig. 5). Iatrogenic redial nerve injury was reported in all the studies. There was no significant difference between the two fixation methods (RR = 0.72 95% CI: 0.35–1.47) (Fig. 6). The incidence of intraoperative fracture comminution was calculated for 6 studies. The available data demonstrated that the incidence of intraoperative fracture comminution was significantly reduced in the DCP groups compared with the IMN groups (RR = 3.14 95% CI: 1.02–9.64) (Fig. 7). The results of the pooled statistical analysis shown in Figure 8 showed that the RR of infection was 0.48 (95% CI, 0.19–1.24), but there was no statistically significant difference found between the groups. From the seven studies reporting shoulder impingement, the increased rate for shoulder impingement in the IMN group was statistically significant compared with the DCP group (RR = 7.32 95% CI: 2.64–20.29) (Fig. 9). Fort studies reported the number of patients who suffered from a restriction of shoulder range of movement. The meta-analysis showed that the patients' shoulder movement after DCP treatment was superior to the patients after IMN fixation (RR = 9.27 95% CI: 2.22–38.72) (Fig. 10). DCP fixation yielded a significantly lower RR of implant failure than nailing fixation (RR = 3.23 95% CI: 1.15–9.06) (Fig. 11). The incidence of re-operation was significantly lower in the DCP group than the IMN group (RR = 2.21 95% CI: 1.28–3.81) (Fig. 12).

### Publication bias

The publication bias test was performed for the overall populations by the incidence of radial nerve injury. No significant publication bias was shown for the overall populations by the Begg rank correlation method (P = 0.107) and Egger weighted regression method (P = 0.992).

### Quality of the evidence and recommendation strengths

Nine outcomes in this meta-analysis were evaluated using the GRADE system. The following 4 outcomes were important: fracture union, ASES score, radial nerve injury, and intraoperative fracture comminution. The evidence quality for each outcome was very low (Table 3). We agreed that the overall evidence quality was very low. This finding may lower the confidence in any recommendations.

**Table 3:** The GRADE evidence quality for each outcome.

Outcome	No of studies	Design	Risk of bias	Inconsistency	Indirectness	Imprecision	Other considerations	No of patients (IMN versus DCP)	No of patients (Control)	Relative effect (95% CI)	Absolute effect	Quality	Importance
Fracture union	10	randomized trials	very serious^1^	no serious inconsistency^1^	no serious indirectness	serious^1^	none	189/215 (87.9%)	208/225 (92.4%)	RR 0.96 (0.9–1.02)	37 fewer per 1000 (from 92 fewer to 18 more)	⊕○○○ VERY LOW	IMPORTANT
								NA	94.4%		38 fewer per 1000 (from 94 fewer to 19 more)		
ASES score	3	randomized trials	very serious^1^	no serious inconsistency	no serious indirectness	serious^1^	reporting bias^1^	57	63	NA	MD 1.84 lower (3.91 lower to 0.22 higher)	⊕○○○ VERY LOW	IMPORTANT
Radial nerve injury	10	randomized trials	very serious^1^	serious^1^	no serious indirectness	no serious imprecision	none	8/215 (3.7%)	13/225 (5.8%)	RR 0.72 (0.35 to 1.47)	16 fewer per 1000 (from 38 fewer to 27 more)	⊕○○○ VERY LOW	IMPORTANT
								NA	4.6%		13 fewer per 1000 (from 30 fewer to 22 more)		
Intraoperative fracture comminution	6	randomized trials	very serious^1^	no serious inconsistency	no serious indirectness	serious^1^	none	10/128 (7.8%)	2/137 (1.5%)	RR 3.14 (1.02 to 9.64)	31 more per 1000 (from 0 more to 126 more)	⊕○○○ VERY LOW	NOT IMPORTANT
								NA	0%		NA		
Infection	9	randomized trials	very serious^1^	serious^1^	no serious indirectness	serious^1^	none	4/205 (2%)	11/212 (5.2%)	RR 0.48 (0.19 to 1.24)	27 fewer per 1000 (from 42 fewer to 12 more)	⊕○○○ VERY LOW	NOT IMPORTANT
								NA	0%		NA		
Shoulder impingment	7	randomized trials	very serious^1^	no serious inconsistency	no serious indirectness	serious^1^	none	24/157 (15.3%)	1/168 (0.6%)	RR 7.32 (2.64 to 20.29)	38 more per 1000 (from 10 more to 115 more)	⊕○○○ VERY LOW	NOT IMPORTANT
								NA	0%		NA		
Restriction of shoulder range of movement	4	randomized trials	very serious^1^	no serious inconsistency	no serious indirectness	serious^1^	none	15/95 (15.8%)	0/104 (0%)	RR 9.27 (2.22 to 38.72)	NA	⊕○○○ VERY LOW	NOT IMPORTANT
								NA	0%		NA		
Implant failure	7	randomized trials	very serious^1^	no serious inconsistency	no serious indirectness	serious^1^	none	11/150 (7.3%)	2/160 (1.3%)	RR 3.23 (1.15 to 9.06)	28 more per 1000 (from 2 more to 101 more)	⊕○○○ VERY LOW	NOT IMPORTANT
								NA	0%		NA		
Re-operation	9	randomized trials	very serious^1^	serious^1^	no serious indirectness	serious^1^	none	34/195 (17.4%)	16/203 (7.9%)	RR 2.21 (1.28 to 3.81)	95 more per 1000 (from 22 more to 221 more)	⊕○○○ VERY LOW	NOT IMPORTANT
								NA	5%		61 more per 1000 (from 14 more to 140 more)		

^1^ No explanation was provided; IMN: intramedullary nailing; DCP: dynamic compression plate.

## Discussion

Fractures of the humeral shaft are common and result in a significant burden to society [1]. Surgical treatment is generally accepted for open fractures, polytrauma patients, ipsilateral humeral shaft and forearm fractures, and cases in which there is a failure to maintain alignment in a functional status [5]. Recent advances in internal fixation have improved the surgical outcomes [23], [29]–[31]. IMN and DCP are alternatives in the treatment of patients with humeral shaft fracture, with each method resulting in relatively high union rates [3], [5]. Several published studies have demonstrated that IMN and DCP improve the preoperative clinical status, but it is not clear which of these two interventions provides better outcomes. In 2011, a Cochrane systematic review[32] was published, which indicated that IMN was associated with an increased risk of shoulder impingement and a related increase in the restriction of shoulder movement and need for removal of the metalwork. Ouyang et al.[33] updated a meta-analysis and reported that both implants could achieve a similar treatment effect on humeral shaft fractures. There has been no consensus regarding whether DCP is an optimal operation compared with IMN. There have been no guidelines or recommendations for surgically treating humeral shaft fractures. There is a need for an evidence base to help surgeons make clinical decisions and develop optimal surgical treatments. To the best of our knowledge, this study is the first meta-analysis that uses the GRADE system to evaluate the quality of the evidence comparing the IMN and DCP treatments for humeral shaft fractures.

Although Kurup et al.[32] conducted a Cochrane systematic review, only five studies were included, which may lower the reliability and stability of the pooled results. Ouyang et al.[33] performed an updated meta-analysis but did not compare the functional outcomes between IMN and DCP. The two previous meta-analyses did not use the GRADE system to assess the quality of the current evidence. The quality of evidence may help the clinicians to guide their decisions and help medical policy makers to make recommendations. Rating the quality of evidence by GRADE was introduced in the present meta-analysis.

The methodological quality assessment identified a number of limitations to the current evidence base. (1) The majority of the RCTs had insufficient information on the randomization methods. Only three included studies used sealed envelopes for allocation concealment. The patients of the two quasi-RCTs were randomly allocated by an incorrect randomization method, namely by odd and even hospital numbers. These methods permitted selection and allocation bias. None of the included studies reported blinding of the surgeons, participants or assessors, allowing for assessor and expectation bias and the potential for type II statistical errors regarding these outcomes. The efficacy of the statistics could be improved in the future by including more RCTs. The studies with a high risk of bias included in this meta-analysis would overestimate the treatment effects. (2) Although RCTs or quasi-RCTs were performed, the relatively small number of participants restricted the statistical power. (3) The long-term follow-up results may change the current conclusions. (4) Clinical heterogeneity may be caused by the preexisting conditions of the patients, various indications for surgeries, the experience level of the orthopedic surgeons, fracture type, medical commodities, smoking and the age of fractures (acute or nonrecent fractures). The above confounding factors might have an impact on the present outcomes. (5) Meta-analyses are subject to bias and provide inappropriate estimates for the effect of treatment when compared to successive large RCTs [34]. It is important to remember that publication bias may exist because the negative results are less likely to be published. While the results of this meta-analysis should be considered appropriate, these methodological defects should be considered when interpreting the findings.

All of the included studies conducted comparisons between IMN and DCP in treating humeral shaft fractures, but no study provided conclusive evidence in favor of either surgical approach. The pooled data for fracture unions did not result in significant findings favoring one of the two implants. IMN provides acceptable micromotion at the fracture site, which promotes fracture healing, but the effects of reaming may exert an influence on the blood supplying at the fracture site. DCP could offer an accurate reduction, which may promote fracture healing. IMN could achieve a comparable fracture union rate to DCP fixation. The timing of delayed union or nonunion varied in the studies of the present meta-analysis, which may increase the risk of bias of the pooled results. A unified timing of fracture union is required in future studies.

Regarding the ASES score, though there was a trend favoring DCP, the present meta-analysis found no statistically significant differences between the two interventions. There was not enough evidence to determine the ASES score between the two groups because only three studies were included in the present meta-analysis. The statistical power may be lower because of the small sample size.

In respect to iatrogenic radial nerve injury, the difference between the two groups was not statistically significant. The open reduction and internal fixation of humeral shaft fractures can be performed through a variety of approaches. The anterolateral approach is useful for the exposure of fractures involving the proximal and middle thirds of the humeral shaft. The benefits of this approach include its extensile nature and avoidance of the radial nerve. A posterior approach may be better suited for fractures extending between the olecranon fossa and the distal middle-third of the humerus. This approach accommodates very distal plating of the humerus along the posterolateral column, where additional fixation into the posterior capitellum is critical for the stability of the distal segment. The anteromedial approach may be of benefit because of the direct access it provides to the radial nerve. The selection of a surgical approach by the surgeons is determined by his or her experience and the location of fractures. In both approaches, the radial nerve must be dissected and identified to avoid iatrogenic injury from either cutting it during exposure or plating over it during fracture fixation. A detailed description of the approach in each patient cannot be obtained from the individual studies. The incidence of radial nerve injury by DCP fixation was affected by the above confounding factors. In the IMN procedure, the iatrogenic stretching of the radial nerve during ante grade nailing with the use of a mallet might account for the iatrogenic radial nerve injury. IMN was comparable to DCP in iatrogenic radial nerve palsies, but the fact that IMN has the advantage of preventing exposure and soft-tissue stripping might make this method preferable over DCP fixation for surgeons.

Regarding iatrogenic fracture comminution, the pooled results showed that DCP was superior to IMN. For the distal third of the humeral shaft, retrograde nailing is indicated only in patients with a wide medullary canal. It is critical to create a sufficiently large entry portal and to perform careful introduction to avoid supracondylar or other additional fractures.[9] Fracture healing would not be affected even if additional comminution occurs at the fracture site.[10] The included studies with ante grade nailing and retrograde nailing were combined in the present analysis without subgroup analysis, which might introduce selection bias to the study.

Regarding the risk of infection, the impairment of the blood supply and wide exposure may increase the risk of infection with DCP, especially in the middle and distal parts of the humeral shaft. Minimally invasive plating osteosynthesis, as a minimal invasive and limited dissection technique, has recently been used to protect the blood supply at the fracture site [35], [36]. The drawbacks of this method, such as difficulty in achieving closed reduction and intraoperative nerve injury, limit its application in clinical practice. In the IMN groups, a risk of infection might be introduced by reaming which may lead to an impairment of blood supply of the medial cortex. The injury severity, patient selection, and postoperative rehabilitation may exert influence on the stability of the pooled results.

The meta-analysis showed that DCP is superior to IMN regarding shoulder impingement and restriction of the shoulder range of motion. In patients with humeral shaft fracture who underwent IMN, shoulder impingement was caused by a prominent nail, and the restriction of the shoulder range of motion was caused by scar tissue and/or damage to the rotator cuff in its critical zone of hypovascularity creating chronic tendon tearing. Several studies have investigated different approaches to improve the outcomes by avoiding the avascular zone of the rotator cuff. These studies reported that careful repair of the tendon after nail insertion may provide better outcomes and less morbidity [37], [38].

In the respect to implant failure and the re-operation rates, the pooled results showed that DCP was superior to IMN. In the DCP groups, the rotation force and shear force were unable to pass through the fracture site, which led to higher stress concentrations on the implant. In the IMN groups, implant failure usually occurred due to breakage of the nail at the site of the distal or proximal locking screw. This complication was frequently associated with patient rehabilitation after fixation. The plans and methods for postoperative rehabilitation should be formulated by the radiographic evaluation of healing, which may lower the risk of implant failure. The re-operation rate was an overall outcome because the presence of complications such as infection, implant failure, restriction of joint motion, nonunion, or delay union usually necessitates a re-operation such as implant exchange or implant removal to achieve refixation or functional recovery. The indications for re-operation for certain complications remained inconclusive [39], [40]. The different indications for re-operation among the individual studies may pose a potential bias with regard to the pooled results.

In the GRADE system of rating the quality of evidence, each RCT began as high quality evidence but was rated down by five categories of limitations. The method of random sequence generation was not stated in most of trials, and insufficient details were provided to confirm adequate concealment of allocation. Variation in the timing of follow-up was a potential source of bias, and an intention-to-treat analysis was not confirmed in any of the trials. There were no independent or blinded assessments of the outcome. We consider that the above factors could significantly influence the stability of the outcomes. The above factors of methodological quality accounted for the downgraded evidence quality. The overall strength of the references was very low. The overall very low evidence quality indicates that the operations should be conducted according to the characteristics of the individual patients in clinical practice. High-quality clinical trials are required to compare the advantages or disadvantages of IMN and DCP in the future. To some extent, the present study is meaningful for both clinical treatment and fundament research.

The present study showed that both IMN and DCP can achieve similar fracture union rates with similar incidences of radial nerve injury and infection. IMN was associated with increased risk of shoulder impingement, a higher restriction of shoulder movement, an increased risk of intraoperative fracture comminution, a higher incidence of implant failure, and an increased risk of re-operation. DCP may be superior to IMN in the treatment of humeral shaft fractures, but the overall quality of the evidence is very low according to the GRADE system, which may reduce our confidence in the recommendations for the use of DCP. Because of the very low quality of evidence, sufficiently sized and methodologically sound RCTs are needed to assess the safety and efficiency of the IMN and DCP procedures in treating humeral shaft fractures.

## Supporting Information

Checklist S1 Preferred reporting items for systematic reviews and meta-analysis (PRISMA) 2009 Checklist.
(DOC)

## References

[1] Tsai CH, Fong YC, Chen YH, Hsu CJ, Chang CH, et al (2009) The epidemiology of traumatic humeral shaft fractures in Taiwan. Int Orthop 33: 463–467. 18414861 10.1007/s00264-008-0537-8 PMC2899047

[2] Sarmiento A, Zagorski JB, Zych GA, Latta LL, Capps CA (2000) Functional bracing for the treatment of fractures of the humeral diaphysis. J Bone Joint Surg Am 82: 478–486. 10761938 10.2106/00004623-200004000-00003

[3] Foster RJ, Dixon GJ, Bach AW, Appleyard RW, Green TM (1985) Internal fixation of fractures and non-unions of the humeral shaft. Indications and results in a multi-center study. J Bone Joint Surg Am 67: 857–864. 4019533

[4] Sarmiento A, Kinman PB, Galvin EG, Schmitt RH, Phillips JG (1977) Functional bracing of fractures of the shaft of the humerus. J Bone Joint Surg Am 59: 596–601. 873955

[5] Sarmiento A, Waddell JP, Latta LL (2002) Diaphyseal humeral fractures: treatment options. Instr Course Lect 51: 257–269. 12064111

[6] Mulier T, Seligson D, Sioen W, van den Bergh J, Reynaert P (1997) Operative treatment of humeral shaft fractures. Acta Orthop Belg 63: 170–177. 9415724

[7] Robinson CM, Bell KM, Court-Brown CM, McQueen MM (1992) Locked nailing of humeral shaft fractures. Experience in Edinburgh over a two-year period. J Bone Joint Surg Br 74: 558–562. 1624516 10.1302/0301-620X.74B4.1624516

[8] Changulani M, Jain UK, Keswani T (2007) Comparison of the use of the humerus intramedullary nail and dynamic compression plate for the management of diaphyseal fractures of the humerus. A randomised controlled study. Int Orthop 31: 391–395. 16900354 10.1007/s00264-006-0200-1 PMC2267584

[9] Rommens PM, Kuechle R, Bord T, Lewens T, Engelmann R, et al (2008) Humeral nailing revisited. Injury 39: 1319–1328. 18417134 10.1016/j.injury.2008.01.014

[10] Rommens PM, Verbruggen J, Broos PL (1995) Retrograde locked nailing of humeral shaft fractures. A review of 39 patients. J Bone Joint Surg Br 77: 84–89. 7822403

[11] Atkins D, Eccles M, Flottorp S, Guyatt GH, Henry D, et al (2004) Systems for grading the quality of evidence and the strength of recommendations I: critical appraisal of existing approaches The GRADE Working Group. Bmc Health Serv Res 4: 38. 15615589 10.1186/1472-6963-4-38 PMC545647

[12] Atkins D, Best D, Briss PA, Eccles M, Falck-Ytter Y, et al (2004) Grading quality of evidence and strength of recommendations. BMJ 328: 1490. 15205295 10.1136/bmj.328.7454.1490 PMC428525

[13] Egger M, Davey SG, Schneider M, Minder C (1997) Bias in meta-analysis detected by a simple, graphical test. BMJ 315: 629–634. 9310563 10.1136/bmj.315.7109.629 PMC2127453

[14] Begg CB, Mazumdar M (1994) Operating characteristics of a rank correlation test for publication bias. Biometrics 50: 1088–1101. 7786990

[15] Guyatt GH, Oxman AD, Vist G, Kunz R, Brozek J, et al (2011) GRADE guidelines: 4. Rating the quality of evidence–study limitations (risk of bias). J Clin Epidemiol 64: 407–415. 21247734 10.1016/j.jclinepi.2010.07.017

[16] Guyatt GH, Oxman AD, Kunz R, Woodcock J, Brozek J, et al (2011) GRADE guidelines: 7. Rating the quality of evidence–inconsistency. J Clin Epidemiol 64: 1294–1302. 21803546 10.1016/j.jclinepi.2011.03.017

[17] Guyatt GH, Oxman AD, Kunz R, Woodcock J, Brozek J, et al (2011) GRADE guidelines: 8. Rating the quality of evidence–indirectness. J Clin Epidemiol 64: 1303–1310. 21802903 10.1016/j.jclinepi.2011.04.014

[18] Guyatt GH, Oxman AD, Kunz R, Brozek J, Alonso-Coello P, et al (2011) GRADE guidelines 6. Rating the quality of evidence–imprecision. J Clin Epidemiol 64: 1283–1293. 21839614 10.1016/j.jclinepi.2011.01.012

[19] Guyatt GH, Oxman AD, Montori V, Vist G, Kunz R, et al (2011) GRADE guidelines: 5. Rating the quality of evidence–publication bias. J Clin Epidemiol 64: 1277–1282. 21802904 10.1016/j.jclinepi.2011.01.011

[20] Guyatt GH, Oxman AD, Sultan S, Glasziou P, Akl EA, et al (2011) GRADE guidelines: 9. Rating up the quality of evidence. J Clin Epidemiol 64: 1311–1316. 21802902 10.1016/j.jclinepi.2011.06.004

[21] Putti AB, Uppin RB, Putti BB (2009) Locked intramedullary nailing versus dynamic compression plating for humeral shaft fractures. J Orthop Surg (Hong Kong) 17: 139–141. 19721138 10.1177/230949900901700202

[22] Benegas E, Amodio D, Correia L, Malavolta A, Ramadan L, et al (2007) Comparative, prospective and randomized study of humeral shaft fractures requiring surgical treatment bridging plate versus locked intramedullary nail (preliminaryanalysis). Acta Ortop Bras 15: 87–92.

[23] Chapman JR, Henley MB, Agel J, Benca PJ (2000) Randomized prospective study of humeral shaft fracture fixation: intramedullary nails versus plates. J Orthop Trauma 14: 162–166. 10791665 10.1097/00005131-200003000-00002

[24] Kesemenli CC, Subasi M, Arslan H, Necmioglu S, Kapukaya A (2003) [Comparison between the results of intramedullary nailing and compression plate fixation in the treatment of humerus fractures]. Acta Orthop Traumatol Turc 37: 120–125. 12704250

[25] McCormack RG, Brien D, Buckley RE, McKee MD, Powell J, et al (2000) Fixation of fractures of the shaft of the humerus by dynamic compression plate or intramedullary nail. A prospective, randomised trial. J Bone Joint Surg Br 82: 336–339. 10813165 10.1302/0301-620x.82b3.9675

[26] Raghavendra S, Bhalodiya HP (2007) Internal fixation of fractures of the shaft of the humerus by dynamic compression plate or intramedullary nail: A prospective study. Indian J Orthop 41: 214–218. 21139747 10.4103/0019-5413.33685 PMC2989121

[27] Bolano L, Iaquinto J, Vasicek V (1995) Operative treatment of humerus shaft fractures: a prospective randomized study comparing intra-medullary nailing with dynamic compression plating [abstract]. In. p. 33.

[28] Li Y, Wang C, Wang M, Huang L, Huang Q (2011) Postoperative malrotation of humeral shaft fracture after plating compared with intramedullary nailing. J Shoulder Elbow Surg 20: 947–954. 21440461 10.1016/j.jse.2010.12.016

[29] Singisetti K, Ambedkar M (2010) Nailing versus plating in humerus shaft fractures: a prospective comparative study. Int Orthop 34: 571–576. 19506868 10.1007/s00264-009-0813-2 PMC2903148

[30] Bhandari M, Devereaux PJ, McKee MD, Schemitsch EH (2006) Compression plating versus intramedullary nailing of humeral shaft fractures–a meta-analysis. Acta Orthop 77: 279–284. 16752291 10.1080/17453670610046037

[31] Martinez AA, Cuenca J, Herrera A (2004) Treatment of humeral shaft nonunions: nailing versus plating. Arch Orthop Trauma Surg 124: 92–95. 14652778 10.1007/s00402-003-0608-7

[32] Kurup H, Hossain M, Andrew JG (2011) Dynamic compression plating versus locked intramedullary nailing for humeral shaft fractures in adults. Cochrane Database Syst Rev:D5959 10.1002/14651858.CD005959.pub2 PMC12597225 21678350

[33] Ouyang H, Xiong J, Xiang P, Cui Z, Chen L, et al. (2012) Plate versus intramedullary nail fixation in the treatment of humeral shaft fractures: an updated meta-analysis. J Shoulder Elbow Surg 10.1016/j.jse.2012.06.007 22947239

[34] LeLorier J, Gregoire G, Benhaddad A, Lapierre J, Derderian F (1997) Discrepancies between meta-analyses and subsequent large randomized, controlled trials. N Engl J Med 337: 536–542. 9262498 10.1056/NEJM199708213370806

[35] Apivatthakakul T, Phornphutkul C, Laohapoonrungsee A, Sirirungruangsarn Y (2009) Less invasive plate osteosynthesis in humeral shaft fractures. Oper Orthop Traumatol 21: 602–613. 20087720 10.1007/s00064-009-2008-9

[36] Apivatthakakul T, Patiyasikan S, Luevitoonvechkit S (2010) Danger zone for locking screw placement in minimally invasive plate osteosynthesis (MIPO) of humeral shaft fractures: a cadaveric study. Injury 41: 169–172. 19735916 10.1016/j.injury.2009.08.002

[37] Dimakopoulos P, Papadopoulos AX, Papas M, Panagopoulos A, Lambiris E (2005) Modified extra rotator-cuff entry point in antegrade humeral nailing. Arch Orthop Trauma Surg 125: 27–32. 15723245 10.1007/s00402-004-0757-3

[38] Park JY, Pandher DS, Chun JY, Md ST (2008) Antegrade humeral nailing through the rotator cuff interval: a new entry portal. J Orthop Trauma 22: 419–425. 18594308 10.1097/BOT.0b013e318173f751

[39] Cole PA, Wijdicks CA (2007) The operative treatment of diaphyseal humeral shaft fractures. Hand Clin 23: 437–448. 18054671 10.1016/j.hcl.2007.11.004

[40] Shao YC, Harwood P, Grotz MR, Limb D, Giannoudis PV (2005) Radial nerve palsy associated with fractures of the shaft of the humerus: a systematic review. J Bone Joint Surg Br 87: 1647–1652. 16326879 10.1302/0301-620X.87B12.16132

